# Bioinformatics Analysis of Immune Cell Infiltration and Diagnostic Biomarkers between Ankylosing Spondylitis and Inflammatory Bowel Disease

**DOI:** 10.1155/2023/9065561

**Published:** 2023-01-05

**Authors:** Xuhong Zhang, Tao Chen, Xian Qian, Xiaojin He

**Affiliations:** ^1^Wuxi Affiliated Hospital of Nanjing University of Chinese Medicine, Wuxi, China; ^2^Nanjing University of Chinese Medicine, Nanjing, China; ^3^Affiliated Hospital of Nanjing University of Chinese Medicine, Nanjing, China

## Abstract

**Background:**

Ankylosing spondylitis (AS) and inflammatory bowel disease (IBD) are both autoimmune diseases, and they often occur together in clinical practice, but the pathogenesis is unclear. This study is aimed at identifying the hub genes and explore the related immune molecular mechanisms between AS and IBD by bioinformatics analysis.

**Methods:**

From the public Gene Expression Omnibus (GEO) database, the AS and IBD datasets (GSE73754, GSE59071, GSE25101, and GSE36807) were obtained. The immune cell infiltration in the peripheral blood tissues of GSE73754 and GSE59071 was assessed using the CIBERSORT algorithm. Then, we used the Weighted Gene Coexpression Network Analysis (WGCNA) to identify the Differentially Expressed Genes (DEGs) related to AS and IBD. Then, the immune genes from the ImmPort database intersected with the DEGs to obtain hub genes. The Gene Ontology (GO) and Kyoto Encyclopedia of Genes and Genomes (KEGG) analyzed the functional correlation of hub genes. Then, hub genes were verified in GSE25101 and GSE36807. The clusterProfiler software and Gene Set Enrichment Analysis (GSEA) were used to conduct functional enrichment and pathway enrichment studies. Finally, the diagnostic efficacy was assessed using Receiver Operating Characteristic (ROC) curve analysis.

**Results:**

The analysis of immune characteristics showed that both AS and IBD were related to immunity, and neutrophils were positively correlated in both diseases. Nine coexpressed genes, including FCGRT, S100A11, IFNGR1, NFKBIZ, JAK2, LYN, PLAUR, ADM, and IL1RN, were linked to immune cells. The GO and KEGG analyses results showed that enrichment analysis was mainly related to cell transport and migration. Finally, the ROC curve was verified with the validation set, and it was found that PLAUR has clinical diagnostic significance and the most excellent specificity and sensitivity, respectively.

**Conclusions:**

PLAUR (uPAR) is a promising biomarker and will be an underlying genetic biomarker for diagnosing AS comorbid IBD. Inflammation and immunological modulation mediated by neutrophil infiltration were important in the development of AS and IBD and may be diagnostic and therapeutic targets.

## 1. Introduction

Ankylosing Spondylitis (AS) is a common chronic immunological disease that causes axial skeleton inflammation [1]. Epidemiological studies have found that the global incidence of AS is between 0.1% and 1.4%, and the incidence in males is higher than in females [2]. Extra-articular symptoms are indicated, with uveitis and psoriasis being among the most common, as well as intestinal inflammation [3]. Intestinal inflammation is the most common accompanying symptom, with an incidence of up to 40%-60% [4]. With the increased time, there is a 5%-20% probability of developing Inflammatory Bowel Disease (IBD) [5].

IBD is an ongoing condition that affects the digestive system, and it may take several forms, the most prevalent of which are ulcerative colitis (UC) and Crohn's disease (CD) [6]. The prevalence of IBD increased from 0.3% to 1.3% during this decade, and the trend is increasing [7]. Patients with IBD typically present with rectal bleeding, diarrhea, abdominal pain, and weight loss [8]. IBD patients frequently experience extraintestinal manifestations (EIMs), which include lesions of the eyes, skin, liver, gallbladder, and blood [9]. The most frequent EIMs, with an incidence of up to 40% [10], are joint lesions, which are more common in CD than in UC [11].

It is common for AS and IBD to coexist. According to certain studies, people with ankylosing spondylitis have an incidence of inflammatory bowel illness ranging from 6% to 14% [12]. In addition, people with inflammatory bowel illness have a 3.7%–4.5% risk of ankylosing spondylitis [13]. According to population-based matched cohort research, people with AS have a lifetime risk of acquiring IBD, and the incidence increases with the duration of the disease [4]. Both AS and IBD are recurring chronic diseases, and regardless of which condition a patient has, their quality of life is greatly diminished [14, 15]. If they are in a later stage of the disease, they may be unable to care for themselves. Also, patients with comorbidities will have to deal with more physical and mental pain, and the high cost of therapy is another major problem.

Although some progress has been made over the decades in managing and treating AS and IBD, the comorbidity pathogenesis is still unclear. The underlying mechanism of AS and IBD is complex and is caused by the interaction of genetic, environmental, and immune factors [16], in which immune factors play an essential role in the entire process. So, finding out how the immune system works is important for early prevention and treatment of AS and IBD, and that is what current research is all about.

As is associated with a variety of immune cells, including CD4+, CD8+ T cells, and macrophages, which produce cytokines, particularly TNF-*α* and TGF-*β*, which are important in the inflammatory process by causing inflammation, fibrosis, and ossification at enthesitis [17]. The pathogenesis of IBD is also associated with the interaction and imbalance of several immune cells, which results in a cascade of immune-mediated inflammation [18]. In summary, immune cells play a vital role in the occurrence and development of comorbidities. Therefore, a systematic approach is urgently needed to evaluate the contribution of immune cells and explore hub genes related to immune cells. In addition, the evaluation of immune cell infiltration and the determination of the variations in the components of infiltrated immune cells are of tremendous importance for elucidating the molecular mechanisms underlying the progression of AS and IBD and developing novel immunotherapy targets. If the right biomarkers can be found, we can use them for prediagnosis and develop new targeted therapies.

With the advent of high-throughput sequencing and microarray technologies in recent years, bioinformatics analysis may now be used to find new genes and biomarkers for various diseases, including autoimmune diseases [19]. CIBERSORT is an analytical tool that utilizes microarray data or RNA-sequencing (RNA-seq) data to evaluate immune cell expression in samples and obtain various immune cell ratios [20]. This study, CIBERSORT, analyzed immune cell infiltration in AS and IBD, respectively. We used the weighted gene coexpression network analysis (WGCNA) to identify the Differentially Expressed Genes (DEGs) related to AS and IBD. And, we enriched DEGs in the Kyoto Encyclopedia of Genes and Genomes (KEGG) and Gene Ontology (GO). The unique gene signatures in AS and IBD were identified. We identified the critical genes related to AS and IBD using Receiver Operating Characteristics (ROC) analysis on the other two datasets. In addition to systematically analyzing the infiltration of AS and IBD immune cells, this study screened for novel and effective diagnostic biomarkers. The results might provide a new method for diagnosing and treating AS and IBD. This article might be the first to look at the shared gene signatures of AS and IBD using a systems biology approach.

## 2. Materials and Methods

### 2.1. GEO Dataset Download

We utilized the terms “Ankylosing Spondylitis” and “Inflammatory Bowel Disease” to search the GEO (https://www.ncbi.nlm.nih.gov/geo/) database for AS and IBD gene expression profiles. The following were the criteria for inclusion: (1) array-based expression profiling or high-throughput mRNA sequencing; (2) case and control groups are required for gene expression profiling; (3) to guarantee that the WGCNA results are accurate, the number of samples in each group should be at least 20. Finally, the four GEO datasets, GSE73754, GSE59071, GSE25101, and GSE36807, were chosen.

The GSE73754 dataset was based on the GPL10558 platform. It had 72 samples, with 52 AS patients in the experimental group and 20 healthy people in the control group. The GSE59071 was based on the GLP6244 platform. It had 116 samples, including 97 patients with UC, 8 patients with CD, and 11 healthy people. Also, the GSE25101 dataset based on GPL6947, which had 16 samples from AS patients and 16 samples from healthy controls, and the GSE36807 dataset based on GPL570, which had 28 samples from IBD patients and 7 samples from healthy controls, were downloaded to estimate the diagnostic biomarkers. The specifics of the data are detailed in Table 1.

### 2.2. Evaluation of Immune Cell Distribution

We calculated the relative percentage and corresponding *P* value of 22 immune cells in blood tissues between AS and IBD using the CIBERSORT program. With this method's help, the normalized gene expression matrix may be transformed into the population of immune cells entering a tissue. LM22 was retrieved from the CIBERSORT website (http://CIBERSORT.stanford.edu/) for use as a reference expression signature. The LM22 signature matrix identified 22 invading immune cell components. CIBERSORT was used to calculate the *P* values and root mean squared errors for each expression file. Only data having a CIBERSORT *P* value less than or equal to 0.05 was filtered and retained for further analysis. The result was immediately included to produce a complete matrix of immune cell subsets. The R packages “corrplot,” “ggplot2,” and “garment” were used to show the CIBERSORT output.

### 2.3. Weighted Gene Coexpression Network Analysis

An approach called WGCNA (weighted gene coexpression network analysis) may find gene modules of high biological significance and examine the relationships between gene networks and illnesses [21]. Therefore, the “WGCNA” package in the R software (4.2.0) was used to design the network. The AS and IBD GEO datasets contained more than 20,000 genes sequenced. The WGCNA was limited to around 5,000 of these genes since the expression levels of the majority of these genes did not differ significantly between samples. We excluded outlier samples from the hierarchical clustering analysis using the “hclust” function of the R programming language. Using the “pickSoftThreshold” function in the WGCNA package, the relevant soft powers (ranging from 1 to 20) were chosen by the standard for a scale-free network. The adjacency matrix was then constructed using the following formula: aij = |Sij| (aij is the adjacency matrix between gene *i* and gene *j*; Sij is the similarity matrix consisting of Pearson's correlation coefficients for all gene pairs: soft power value). In addition, a hierarchical clustering dendrogram was constructed, and related gene expressions were separated into several modules. Each module was assigned a distinct hue, and the minimum value was set at thirty. The Module Eigengene (ME) was then used to summarize the expression patterns of each module, and the association between ME and clinical characteristics was computed. Thus, the modules with a high correlation coefficient with clinical characteristics were prioritized, and the genes in these modules were chosen for further analysis. In this investigation, the WGCNA soft threshold for AS was 9, and for IBD, it was 6. The other settings were network type = “unsigned,” minModuleSize =20, mergeCutHeight =0.25, and deep split =2.

### 2.4. Screening of Hub Genes and Functional Enrichment Analysis

Modules relevant to the AS and IBD groups were chosen for inclusion. Then, the genes in the highly expressed modules of the two groups of diseases were intersected with the immune-related genes obtained from the ImmPort database (https://www.immport.org/shared/genelists/) to acquire hub genes [22]. Finally, the “clusterProfiler” [23] package was used to perform Gene Ontology (GO) and Kyoto Encyclopedia of Genes and Genomes (KEGG) analyses on the hub genes.

### 2.5. Validate Hub Genes

To validate the shared and unique genes, we used hub genes analysis on other AS and IBD datasets (GSE25101 and GSE36807). Therefore, the Pearson correlation between the hub genes and each of the 22 immune cells was computed, and the result was shown using the “ggplot2” R package. At last, we confirmed the expression levels of hub genes between patients with AS and normal controls, IBD and normal controls in validated datasets, and represented them using box plots.

### 2.6. Gene Set Enrichment Analysis (GSEA)

The “clusterProfiler” R package was used to analyze GO terms and KEGG pathways to determine the likely function of the primary gene (*P* value < 0.05). Gene Set Enrichment Analysis (GSEA) is a method that compares gene enrichment to a specific gene set. This method uses microarray data that was obtained via genome-wide expression profiling [24]. According to PLAUR expression level, gene expression data (including AS and IBD) was split into two groups, PLAUR high and PLAUR low. The molecular signature database (MsigDB) was used for GSEA, and the signature gene set was used for this investigation. Significant enrichment was determined as a gene set that met the following criteria: |NES| > 1, *P* value < 0.05, and FDR < 0.20.

### 2.7. Analysis of the Predictive Value of Biomarkers

A ROC analysis was carried out to evaluate the diagnostic performance. In the GSE25101 and GSE36807 datasets, the area under the ROC curve (AUC) was used to measure the diagnostic performance in distinguishing AS and IBD from control samples.

## 3. Results

### 3.1. GEO Information

The four GEO datasets, GSE73754, GSE59071, GSE25101, and GSE36807, were chosen based on the previously established criteria. Table 1 summarizes the information from the four datasets, such as GSE number, detection platforms, and samples. For the WGCNA, we used GSE73754 and GSE59071 as “discovery cohorts,” and for the DEGs study, we used GSE25101 and GSE36807 as “verified cohorts.”

### 3.2. Immune Cell Infiltration in AS and IBD

**Figure 1:**
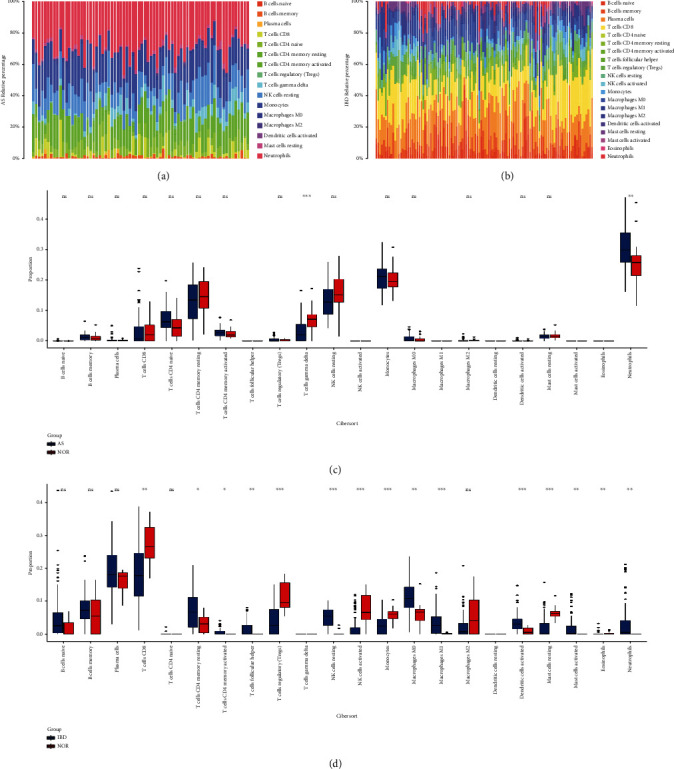
Immune cell infiltration in AS and IBD. (a, b) The bar plot visualizing the relative percent of 22 immune cell in GSE73754 and GSE59071. (c, d) Box diagram of all 22 immune cells differentially infiltrated fraction in GSE7354 and GSE59071 ( ^∗^ for *P* < 0.05,  ^∗∗^ for *P* < 0.01, and  ^∗∗∗^ for *P* < 0.001).

The GSE73754 and GSE59071 samples were used to investigate immune cell infiltration. In AS and IBD, a histogram indicated the composition of 22 immune cells (Figures 1(a) and 1(b)). The colour in the histogram shows the proportion of distinct immune cells in each sample, and the total is 1. The results showed that in AS disease, B cells naïve, monocytes, NK cells resting, T cells gamma delta, T cells CD4 memory activated, T cells CD4 memory resting, and T cells CD8 were the primary infiltrating immune cells. In IBD disease, macrophages M0, T cells follicular helper, T cells CD4 memory activated, T cells CD8, plasma cells, and B cells naïve were the primary infiltrating immune cells. B cells naïve, T cells CD4 memory activated, and T cells CD8 were present in both diseases, indicating a correlation in the mechanism of immune infiltration of the two conditions. The Wilcoxon test was then utilized to determine the significantly different immune cell infiltrates in the AS and control groups as well as the IBD and control groups. The findings of the Wilcoxon test of AS are given as a box diagram in Figure 1(c), this diagram exhibited two different kinds of immune cells with *P* < 0.05. In the AS group, compared with the control group, neutrophils were markedly higher in AS whole blood RNA, while T cells gamma delta was significantly lower in AS whole blood RNA. The findings of the Wilcoxon test of IBD as a box diagram in Figure 1(d). This box diagram exhibited 15 different immune cells with *P* < 0.05. In the IBD group, compared with the control group, T cells CD4 memory resting, T cells CD4 memory activated, T cells follicular helper, NK cells resting, monocytes, macrophages M0, macrophages M1, dendritic cells activated, mast cells activated, eosinophils, and neutrophils were markedly higher in IBD colon tissue. In contrast, T cells CD8, T cells regulatory, NK cells activated, and mast cells resting were significantly lower in IBD colon tissue. Cross-referencing the results of the two figures, only neutrophils were markedly higher in both diseases.

### 3.3. The Coexpression Modules in AS and IBD

**Figure 2:**
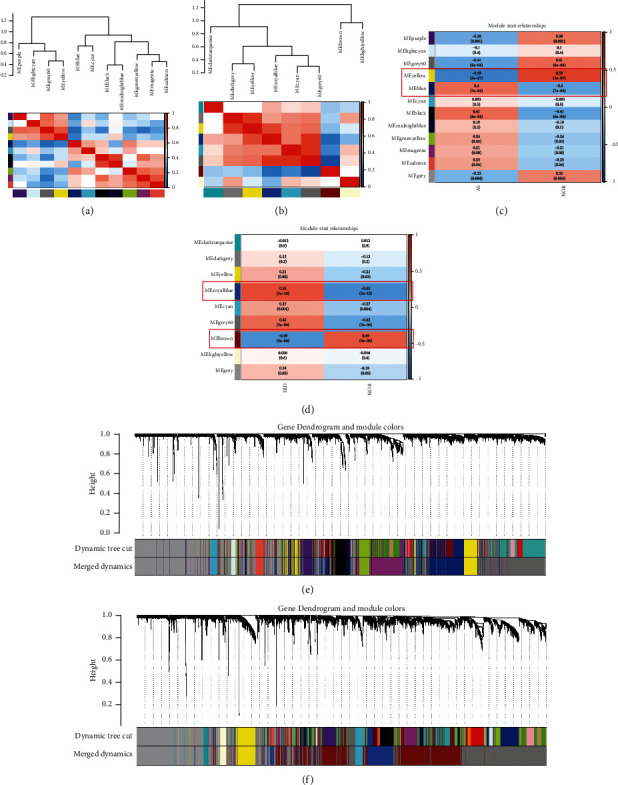
WGCNA in AS and IBD. (a, b) Sample dendrogram and trait heat map in AS and IBD. (c, d) The correlation heat map between gene modules and phenotypes in AS and IBD. (e, f) The coexpression module was established with AS and IBD.

WGCNA in GSE73754 detected 12 modules in the AS illness category, with each hue indicating a separate module. Then, using the Spearman correlation coefficient, a heat map of module-trait connections was created to assess the link between each module and the illness (Figures 2(a), 2(c), and 2(e)). Two modules, “blue” and “yellow,” had a strong relationship with AS and were chosen as AS-relating modules (blue module: *r* = 0.4, *P* = 7*e* − 04; yellow module: *r* = −0.59, *P* = 2*e* − 07). The blue module, which includes 691 genes, was positively linked with AS. The yellow module, which included 299 genes, was negatively linked with AS. Similarly, WGCNA detected 9 modules in GSE59071 for the IBD illness group, with each hue indicating a separate module. Two modules, “brown” and “royal blue” have a high association with IBD and were selected as IBD-related modules (brown module: *r* = −0.49, *P* = 4*e* − 08; royalblue module: *r* = 0.55, *P* = 3*e* − 10). The brown module was negatively correlated with IBD and included 1364 genes. The royal blue module was positively correlated with IBD and included 450 genes (Figures 2(b), 2(d), and 2(f)). Thus, we obtained Differentially Expressed Genes (DEGs) associated with the two diseases (AS and IBD).

### 3.4. Identification of DEGs and Functional Enrichment Analysis

**Figure 3:**
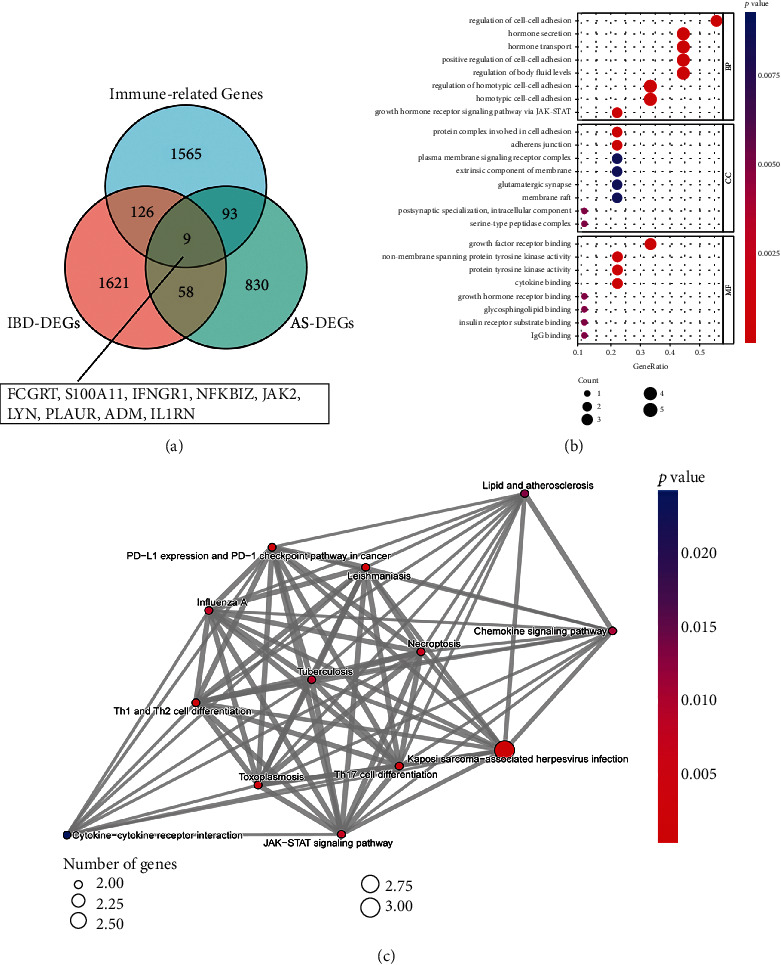
The identification of DEGs and functional enrichment analysis. (a) Venn diagram showing the intersection of hub genes (FCGRT, S100A11, IFNGR1, NFKBIZ, JAK2, LYN, PLAUR, ADM, and IL1RN). (b) BP, CC, and MF analyses in DEGs, the significance of enrichment gradually increases from blue to red, and the size of the dot represents the number of genes contained in the corresponding pathway. (c) KEGG pathway analysis in DEGs, the significance of enrichment gradually increases from blue to red, and the size of the dot represents the number of genes contained in the corresponding pathway.

Through the WGCNA method, 990 DEGs were obtained in the AS disease group, and 1814 DEGs were obtained in the IBD disease group. By matching 2484 IRGs from the ImmPort database, nine hub genes were identified, including FCGRT, S100A11, IFNGR1, NFKBIZ, JAK2, LYN, PLAUR, ADM, and IL1RN (Figure 3(a)). GO and KEGG analyses were performed on hub genes. The GO annotations of hub genes were divided into three sections, Cellular Component (CC), Biological Process (BP), and Molecular Function (MF), which were utilized to examine the functional enrichment of hub genes. For CC, hub genes were chiefly associated with hormone secretion, hormone transport, positive regulation of cell−cell adhesion, and regulation of body fluid levels. The BP included a protein complex involved in cell adhesion and adherens junction. Lastly, for MF, hub genes were primarily interested in growth factor receptor binding, nonmembrane spanning protein tyrosine kinase activity, protein tyrosine kinase activity, cytokine binding, etc. KEGG analysis showed that DEGs were primarily enriched in the Kaposi sarcoma−associated herpesvirus infection, Th17 cell differentiation, PD−L1 expression, and PD−1 checkpoint pathway in cancer, necroptosis, JAK−STAT signaling pathway, etc. (Figures 3(b) and 3(c)).

### 3.5. Validated the Hub Genes

**Figure 4:**
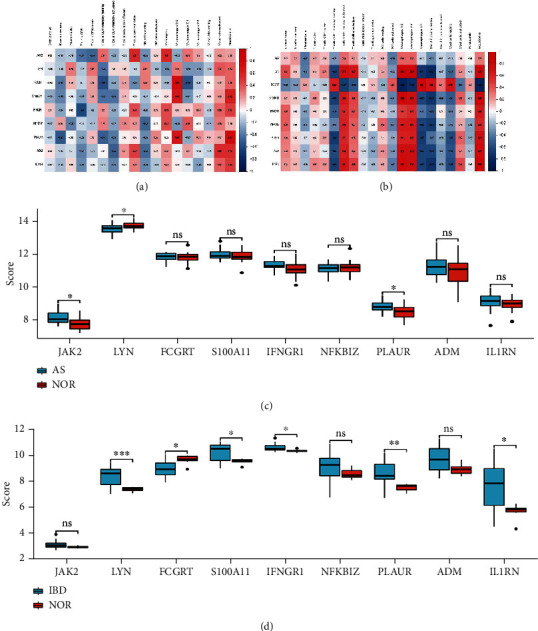
Validated the hub genes. (a, b) The abundance ratios of 22 types of immune cells in GSE25101 and GSE36807 datasets. Red represents a positive correlation, and blue represents a negative correlation. (c, d) 9 hub genes were performed the expression validation used the GSE25101 and GSE36807 datasets (^∗^ for *P* < 0.05, ^∗∗^ for *P* < 0.01, and ^∗∗∗^ for *P* < 0.001).

We validated the correlation between hub genes (FCGRT, S100A11, IFNGR1, NFKBIZ, JAK2, LYN, PLAUR, ADM, and IL1RN) and the abundance ratios of 22 types of immune cells in the GSE25101 and GSE36807 datasets. We discovered many hub genes in the GSE25101 dataset that were associated with the abundance ratio of different immune cells. For example, the level of PLAUR was favorably connected to the abundance ratio of neutrophils, but the level of IFNGR1 was negatively related to the abundance ratio of T cells CD8 (Figure 4(a)). In the GSE36807 dataset, we found that the level of LYN was positively related to the abundance ratio of macrophages M0 and M1. In contrast, the level of FCGRT was inversely associated with the abundance ratio of macrophages M0 and neutrophils (Figure 4(b)). These findings suggested that hub genes may be important in immune cell activity. Then, nine hub genes were performed for expression validation using the GSE25101 and GSE36807 datasets. The expression of LYN was downregulated, and PLAUR and JAK2 were upregulated in patients with AS compared with the control group (Figure 4(c)). FCGRT was downregulated, but LYN, S100A11, PLAUR, IFNGR1, and IL1RN were increased in IBD patients relative to the control group (Figure 4(d)). The findings demonstrated that PLAUR was elevated in both illnesses. So, PLAUR may be a crucial gene.

### 3.6. Gene Set Enrichment Analysis of Key Genes

**Figure 5:**
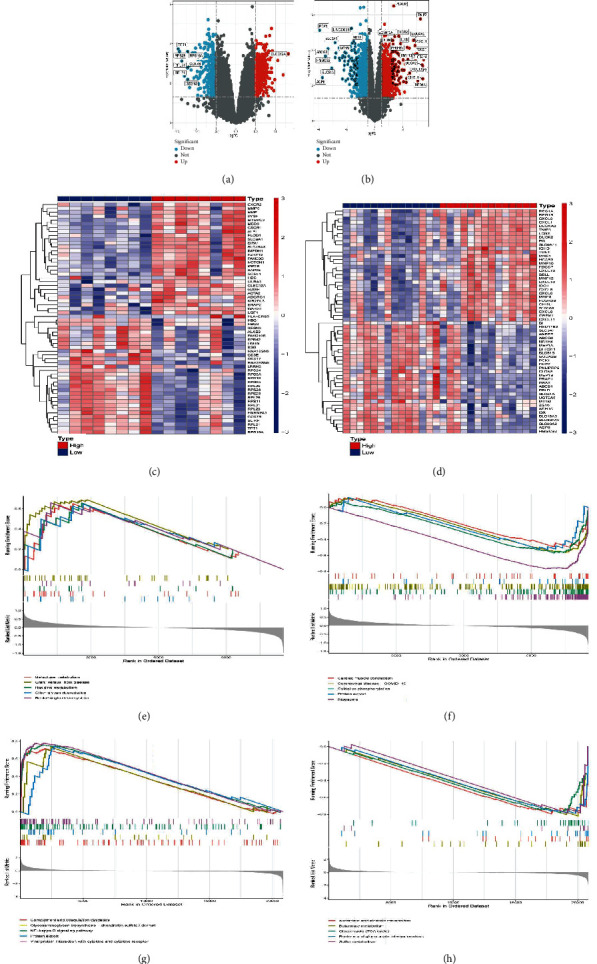
Gene set enrichment analysis of key genes. (a, b) Volcano plots to elucidate the DEGs in AS and IBD samples (adjusted *P* value < 0.05 and |logFC| > 1.2). (c, d) Heat maps depicted the top 30 significantly upregulated DEGs between the PLAUR high and PLAUR low expression groups. Red indicated that the expression of genes was relatively upregulated, and blue indicated the expression of genes was relatively downregulated. (e) The signal pathway enriched in PLAUR high expression groups in AS. (f) The signal pathway enriched in PLAUR low expression groups in AS. (g) The signal pathway enriched in PLAUR high expression groups in IBD. (h) The signal pathway enriched in PLAUR low expression groups in IBD.

To investigate the precise function and downstream pathways generated by PLAUR expression, based on the GSE25101 and GSE36807 datasets, we built two volcano plots to illustrate the DEGs in AS and IBD samples between the PLAUR high and low groups. 273 protein-coding genes were elevated, and 250 were downregulated in the AS disorders category (Figure 5(a)). In the IBD disorders category, there were 797 upregulated and 726 downregulated protein-coding genes (Figure 5(b)). In addition, heat maps illustrated the top 30 DEGs substantially elevated between the PLAUR high and low expression groups (Figures 5(c) and 5(d)). Last, the PLAUR-related genes were further examined using GSEA to discover signaling pathways enriched in AS and IBD. The result showed that GSEA in AS of differentially expressed genes revealed five positively regulated hallmark signatures galactose metabolism, graft−versus−host disease, histidine metabolism, other glycan degradation, renin−angiotensin system (Figure 5(e)), and five negatively regulated hallmark signatures were cardiac muscle contraction, coronavirus disease−COVID−19, oxidative phosphorylation, protein export, and ribosome (Figure 5(f)). GSEA in IBD of differentially expressed genes revealed five positively regulated hallmark signatures, complement and coagulation cascades, glycosaminoglycan biosynthesis−chondroitin sulfate/dermatan sulfate, NF − kappa B signaling pathway, protein export, and viral protein interaction with cytokine and cytokine receptor (Figure 5(g)). Five negatively regulated hallmark signatures were ascorbate and alternate metabolism, butanoate metabolism, citrate cycle (TCA cycle), pentose and glucuronate interconversions, and sulfur metabolism (Figure 5(h)).

### 3.7. ROC Curve Analysis

**Figure 6:**
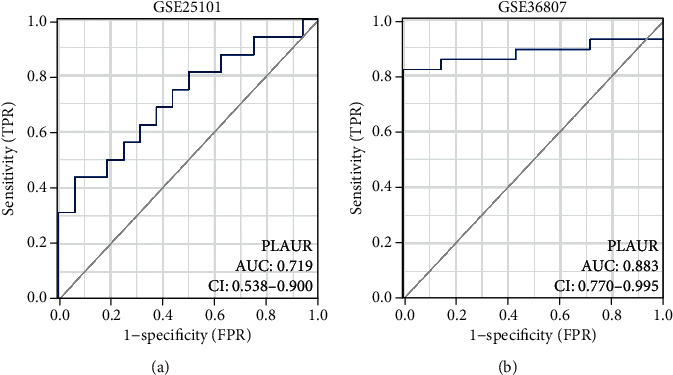
ROC curve analysis. (a) The AUC of PLAUR was 0.719. (b) The AUC of PLAUR was 0.883.

ROC curve analyses were used to assess the discriminatory ability of the PLAUR in AS and IBD datasets (GSE25101 and GSE36807). The AUC of PLAUR was 0.719 in GSE25101 (Figure 6(a)) and 0.883 in GSE36807 (Figure 6(b)). It had diagnostic significance.

## 4. Discussion

Ankylosing spondylitis and inflammatory bowel disease are systemic, progressive, and recurrent chronic diseases [25, 26]. According to epidemiology, the incidence of both diseases is increasing [2, 7]. No matter what type of disease it is, it will seriously affect patients' quality of life, not to mention patients with comorbidities. There is currently no complete cure for patients with AS or IBD, and the mechanism of the link between AS and IBD is unclear. Therefore, exploring the molecular mechanisms between AS and IBD, early identification, and intervention undoubtedly have important clinical significance. According to earlier research, AS and IBD own have their unique genetic inheritance [27, 28], and the activity of the two diseases affects one another [29, 30]. As a result, scientists focus primarily on genetics to investigate the comorbidity mechanism of the two illnesses. At present, the articular peptide hypothesis and the unfolded protein response (UPR) hypothesis are recognized by most researchers as pathogenic mechanisms [31, 32]. While genetic research has been carried out in great detail, it can only account for a tiny portion of the pathogenesis [33]. The development of genome wide association studies (GWAS) and the identification of a large number of genetic variants have made up for the lack of genetic research, revealing the important correlation between non-MHC genes and pathogenic factors [34]. Now that next-generation sequencing and gene chip technologies are available, researchers can find the differentially expressed genes that cause diseases to start and get worse. Because of this, it is helpful to combine the expression data from the microarray with complete bioinformatics analysis. In previous bioinformatics studies, researchers have mainly analyzed a single disease. Meng et al. [35] found the hub genes, comprising MRPL13, MRPL22, LSM3, COX7A2, COX7C, EP300, PTPRC, and CD4, which could be the treatment targets in AS. Cheng et al. [36] found that GNG11, GNB4, AGT, PIK3R3, and CCR7 are key genes that have the significance of distinguishing biomarkers in IBD disease.

Immunity and inflammation are the main factors affecting the activity and development of AS and IBD diseases. To test this conclusion, we extensively used CIBERSORT to detect immune infiltration in AS and IBD and to identify the function of immune cell infiltration in AS and IBD. Using this method, we tried to identify shared immune-related genes in these two diseases, providing a new route for immunotherapy and disease detection. According to the results, neutrophils were positively correlated in both diseases. Neutrophils play a role in various illnesses, including infection, cardiovascular disease, inflammatory disorders, and cancer, making them appealing therapeutic targets [37]. Some researchers [38] believe elevated Tim-3 expression on neutrophils may be a unique marker for assessing disease activity and severity in AS. It may work as a negative feedback mechanism, reducing potential tissue damage caused by excessive inflammatory responses in AS patients. Papagoras et al. [39] provided new insight into the involvement of neutrophils in AS etiology. As a result, neutrophils/NETs were substantial suppliers of IL-1*β* and IL-17, which play critical regulatory roles in AS-related inflammation and new bone formation. A recent study found that CARD9 expression in neutrophils protects against colitis caused by DSS but not in epithelial or CD11c+cells. Without CARD9, mitochondrial dysfunction increases the production of reactive oxygen species, which causes neutrophils to die early through apoptosis, especially in an oxidative environment. The fact that there are fewer working neutrophils in the tissues could explain why fungi are harder to stop, and the risk of intestinal inflammation is higher [40]. There were also reports that the severity of AS was strongly connected to neutrophils [41, 42]. Recently, Zhou et al. found that in addition to neutrophils, monocytes were also associated with AS infiltration and identified LYN as a hub gene [43]. A bioinformatics study on ulcerative colitis discovered that neutrophils were crucial in immune infiltration, B cells, *γδ* T cells, activated mast cells, and M1 macrophages were also involved [44]. According to a recent report, neutrophils are an integral part of the body's innate immune system and play an essential role in maintaining intestinal homeostasis and developing diseases [45]. Increasing neutrophils infiltration also led to microbial dysbiosis, worsened intestinal structural damage, slowed the healing of intestinal inflammation, and increased the risk of thrombosis during IBD [46]. These were consistent with our study.

Differentially expressed WGCNA identified gene modules between the disease group and the control group. By matching genes from the ImmPort database, 9 coexpressed genes were identified (including FCGRT, S100A11, IFNGR1, NFKBIZ, JAK2, LYN, PLAUR, ADM, and IL1RN). The GO and KEGG analyses were conducted to acquire a deeper understanding of the molecular mechanism of these nine genes. The results of GO analyses showed that enrichment analysis was mainly related to cell transport, cell migration, and cell adhesion. Some scholars have found that inflammation-induced aberrant expression of tenascin-C by fibroblast-specific protein-1 (FSP1)+fibroblasts promotes entheseal new bone formation by suppressing extracellular matrix adhesion forces and activating the Hippo signaling [46]. In an inflammatory state, intestinal mucosal vasculature will change, and inflammatory factors will induce vasodilation and congestion, leading to increased vascular permeability, increased translocation of microbial products, and ultimately may increase distant joint inflammation. This migration is dependent on various adhesion molecules and receptors, such as *α*4*β*7 integrin, vascular adhesion protein-1 (VAP-1) [47], and intracellular adhesion molecule-1 (ICAM-1/CD54) [48]. According to one study, cell migration to the gut mucosa may be altered in inflammatory bowel disease, and *α*4*β*7+T cells may upregulate *α*E*β*7 in response to TGF-*β* once inside the gut mucosa [49]. Our conclusion seemed to be similar to these studies.

The KEGG pathway was linked to Kaposi's sarcoma-associated herpesvirus infection, JAK-STAT signaling, Th17 cell differentiation, toxoplasma, Th1 and Th2 cell differentiation, tuberculosis, necroptosis, influenza A, PD−L1 expression, and PD−1 checkpoint pathway in cancer. Previous studies have shown that the increased risk of AS is indeed associated with infections such as *Klebsiella pneumoniae*, tuberculosis, and viruses [50]. It was well recognized that the pathogenesis of IBD was closely related to infection. In addition to the common sources of infection, the human papillomavirus (HPV) and IBD have recently been found to be associated [51]. Both illnesses have a role in pathways involving JAK−STAT signaling and cell differentiation. These were consistent with some previous studies. Cytolethal distending toxin B promotes the development of colitis by eliciting an inflammatory response and activating the JAK-STAT signaling pathway [52]. JAK−STAT signaling was one of the primary pathways investigated in the treatment of AS [53]. In AS, the Th17 and Th2 pathways have opposing regulatory functions [54], and elevated Th17 cells are also associated with cardiovascular problems [55]. In IBD, Th1 cells degrade IEC programming by inducing IEC death, attracting immune cells, increasing IEC adhesion molecule expression, and developing into epithelial cell adhesion molecule–specific interferon *γ*–positive Th1 cells. Pathogenic Th17 cells destroy IECs by activating their IBD susceptibility genes. Colitis may be caused by Th1 and pathogenic Th17 cells [56]. Besides, The T helper cell 17 pathways have emerged as a significant driver of ankylosing spondylitis, rheumatoid arthritis, and inflammatory bowel disease pathogenesis and is a good treatment target [57, 58].

By examining both groups of diseases, we found an essential gene: PLAUR (uPAR). PLAUR (uPAR), a glycoprotein with a molecular mass of 55-60 kD (1 kD = 1 U) and 283 amino acid residues, is linked to the cell membrane by glycosyl-phosphatidyl inositol (GPI) protein. Its gene is found at 19q13, and its DNA is 23 KB long, with 7 exons and 6 introns. uPAR belongs to the lymphocyte antigen 6 (Ly6) superfamily, and its distinctive areas are Ly6 and uPAR (LU). uPAR is made up of three LU sections (D1 to D3) that are linked by short connecting regions and merged to produce a groove structure. Its N-terminal portion in the D1 region may serve as a binding site for uPA, which binds to uPA's N-terminal growth factor domain (GFD). Because uPAR does not have any transmembrane or intracellular domains, it must bind to different transmembrane proteins on the cell surface, like integrin, G protein-coupled receptors, and coreceptors like caveolin, to form functional transmembrane units and then start intracellular signal transduction. The downstream signal transduction pathways involved in uPAR include MAPK, FAK, JAK-STAT, and PI3K-Akt. UPA is the most important ligand of uPAR. The combination of uPA and uPAR can regulate the activity of the plasminogen activation system and hydrolytic ECM proteins and promote cell migration, proliferation, and survival [59]. Some recent studies found that PLAUR was essential in regulating dermatomyositis-interstitial lung disease by neutrophil-associated immune response [60]. PLAUR is a crucial gene in chronic pruritus and functions through E1-PLAUR signaling [61]. Besides, one animal study showed that PLAUR played negative feedback regulation of glucocorticoid pathways in rats' ocular tissues [62]. Some scholars have found that PLAUR facilitated paclitaxel resistance and endometrial cancer cell invasiveness [63]. When epithelial cells moved and grew back when PLAUR was present, it made IBD less likely to happen [64].

GSEA using PLAUR as a single gene suggested that KEGG pathways in the two diseases were mostly related to glycosaminoglycan and metabolism. AS patients' lumbar spines have significant glycosaminoglycan depletion [65, 66], and sulfated glycosaminoglycans have been identified as a new marker for the diagnosis of IBD [67].

## 5. Highlight and Limitations

The highlight of this paper is that it may be the first bioinformatics analysis paper on ankylosing spondylitis and inflammatory bowel disease. The research methods include WCGNA, CIBERSORT, and GSEA. However, there are some limitations to this study. Firstly, the sample of this study lacked clinically relevant information, including comprehensive information on family history and prognosis, disease activity, and disease drug use. Secondly, whether the key gene identified in this study can be used in clinical practice needs to be further verified in clinical and basic trials. Therefore, we will continue to pay attention to PLAUR for subsequent relevant studies.

## 6. Conclusion

Evaluation of immune cell distribution suggested that both AS and IBD were significantly associated with immunity, and the expression of neutrophils was positively correlated in both diseases. In addition, through differential analysis, we identified that PLAUR might be a key gene for the association of the two diseases. This gene has clinical diagnostic significance, which was verified by ROC curve analysis. In conclusion, we speculate that PLAUR might promote the development of both diseases through neutrophils, and the mechanism may be related to transport and migration. This study provided a new reference for further explaining the reasons for the comorbidity of AS and IBD, early diagnostic strategies, prognostic markers, and identifying potential therapeutic targets.

## Figures and Tables

**Table 1:** A summary of the four GEO datasets involving patients with AS and IBD.

ID	GSE number	Platform	Samples	Disease	Group
1	GSE73754	GPL10558	52 patients and 20 controls	AS	Discovery cohort
2	GSE59071	GPL6244	105 patients and 11 controls	IBD	Discovery cohort
3	GSE25101	GPL6947	16 patients and 16 controls	AS	Validation cohort
4	GSE36807	GPL96	28 patients and 7 controls	IBD	Validation cohort

## Data Availability

Publicly available datasets (GSE73754, GSE59071, GSE25101, and GSE36807) were analyzed in this study. All the datasets were obtained from the GEO (http://www.ncbi.nlm.nih.gov/geo/) database.

## Abbreviations

AS: Ankylosing spondylitis
IBD: Inflammatory bowel disease
GEO: Gene Expression Omnibus
WGCNA: Weighted gene coexpression network analysis
DEGs: Differentially expressed genes
GO: Gene Ontology
KEGG: Kyoto Encyclopedia of Genes and Genomes
GSEA: Gene set enrichment analysis
ROC: Receiver operating characteristic
CD: Crohn's disease
UC: Ulcerative colitis
RNA-Seq: RNA-sequencing
NK: Natural killer
ME: Module eigengene
MsigDB: Molecular signatures database
CC: Cellular component
BP: Biological process
MF: Molecular function.
